# Behavioral problems and their relationship to maternal depression, marital relationships, social skills and parenting

**DOI:** 10.1186/s41155-020-00160-x

**Published:** 2020-09-25

**Authors:** Alessandra Turini Bolsoni-Silva, Sonia Regina Loureiro

**Affiliations:** 1 Department of Psychology, School of Sciences, São Paulo State University , Av. Eng. Luiz Edmundo Carrijo Coube, 14-01 - Vargem Limpa, 17033-360, Bauru, São Paulo, Brazil; 2 Department of Neurosciences and Behavioral Sciences, University of São Paulo at Ribeirão Preto, Medical School, Rua Tenente Catão Roxo, 2650, Vila Monte Alegre, 14051-140, Ribeirão Preto, São Paulo, Brazil

**Keywords:** Behavioral problems, Maternal depression, Parenting practices, Marital relationship, Social skills

## Abstract

Behavioral problems have been associated with multiple variables; however, studies simultaneously investigating parenting practices, marital relationships in bi-parental families, maternal depression, and child behavior remain a gap in the literature. The objective was to verify associations between positive and negative parenting practices, marital relationships, social skills, and behavioral problems among children from bi-parental families with and those without maternal depression; to identify the predictive effect of positive and negative parenting practices, marital relationships, children’s social skills, and maternal depression, for internalizing, externalizing behavior problems and internalizing and externalizing comorbidities. A case-control study with a cross-sectional design was adopted to ensure the groups were homogeneous in regard to the children’s, mothers’, and families’ sociodemographic characteristics. A total of 35 mothers currently with depression and 35 without depression indicators participated in the study, while the children were 25 preschoolers and 23 school-aged children. The mothers responded to instruments addressing depression, child behavior, parenting practices, and marital relationships. The results reveal maternal depression associated with marital relationships, positive parenting, and context variables. Maternal depression and marital relationship were found to influence externalizing problems; maternal depression, child-rearing practices, marital relationships, and the children’s behavioral repertoires influence internalizing and externalizing comorbidities; and none of the independent variables influenced the occurrence of internalizing problems.

Behavioral problems are one of the main complaints leading parents to seek psychological assistance for their children. If left untreated, behavior problems may become chronic, with negative consequences over the long term (Goodman, Joyce, & Smith, 2011).

Behavioral problems may be identified as externalizing (disobedience and aggressiveness) or internalizing problems (shyness, anxiety, depression) (Achenbach & Rescorla, 2001). De Los Reyes et al. (2015) conducted an extensive meta-analysis including 341 studies published between 1989 and 2014 (De Los Reyes et al., 2015) that addressed multiple informants and reported that parents and teachers identified externalizing and internalizing problems, as well as comorbidities, with rates varying according to the context (family or school).

Behavioral problems are a multi-determined construct (Alvarenga, Oliveira, & Lins, 2012) and their occurrence is related both to variables that concern children, such as sex, education, and temperament, and the families’ sociodemographic variables like income, education, and family configuration (single-parent or bi-parental families).

In terms of family configurations, having both parents present in the day-to-day routine is known to be a supportive factor for families and a protective factor for children, as a condition that favors more adaptive behavior and better academic outcomes (Leme & Marturano, 2014), as children are less frequently exposed to risk events (Crestani, Mattana, Moraes, & Souza, 2013) when both parents share tasks and care provided to children (Manfroi, Macarini, & Vieira, 2011). This enables greater functionality and communication among family members (Cerveira, 2015).

Merely having the presence of both parents in the family environment, however*,* does not ensure harmonious coexistence, so marital relationship is also a variable to take into account. The systematic literature review conducted by Hameister, Barbosa and Wagner (2015) addresses marital conflict and its repercussions on parenting and child development based on the analysis of 76 papers. It reports that children of couples with difficulties managing marital conflicts were subject to the worst parenting practices, presented the poorest emotional regulation, faced difficulties properly dealing with conflict, and more frequently presented indicators of anxiety and depression. Marital relationship was also addressed in the study by Fantinato and Cia (2015) intended to correlate marital relationship variables, social skills, parents’ social child-rearing skills, and preschoolers’ behaviors, reporting a relationship between the parents’ poor repertoire of social child-rearing skills and negative marital relationships with children’s behavior problems.

The quality of marital relationships and their associations with parenting and child behavior have been addressed by several studies, such as (a) associations between marital conflicts, negative parenting practices, and behavioral problems (Fantinato & Cia, 2015; Hameister, Barbosa, & Wagner, 2015; Ramirez Castillo, 2007; Rhoades, 2008); (b) marital relationships related to parenting (Kanoy, Ulku-Steiner, Cox, & Burchinal, 2003); (c) associations between marital conflict and child adjustment (Schoppe-Sullivan, Schermerhorn, & Cummings, 2007); (d) marital conflicts predicted children’s inferior social competence in terms of coping strategies and/or adjustment (Shelton & Harold, 2007); and (e) the presence of marital conflicts was associated with family members’ mental health problems, including depression (Hsiao, 2017; Najman et al., 2014; Neves & Duarte, 2015).

Another situation impacting families and child behavior involves mental health problems, especially maternal depression, which is a highly prevalent condition (WHO, 2017) that, given its typical symptomatology (American Psychiatric Association [APA], 2014), may affect an individual’s ability to raise children properly.

Pizeta, Silva, Cartafina, and Loureiro (2013) reviewed 68 papers addressing associations between maternal depression, mental health, and child behavior, concluding that (a) maternal depression is associated with internalizing problems, especially in the presence of other risks, among which is negative parenting style; (b) negative parenting style, coupled with maternal depression, is associated with conduct problems and externalizing behavior, especially when depression is severe and chronic; and (c) maternal depression is associated with children’s difficulty to socially interact with family members and peers. The authors report that maternal depression is a risk condition for the development of child behavior problems, internalizing problems, mainly among girls, and externalizing problems among school-aged children and adolescents. Studies report that family interactions are relevant variables associated with maternal depression and child behavior, indicating a need to understand multiple associated factors, including protective factors.

Recently, Shaw, Sitnick, Reuben, Dishion, and Wilson (2016) conducted a longitudinal study monitoring boys from low-income families, from early childhood to adolescence, and verified that behavioral problems coupled with maternal depression present reciprocal and independent effects and that both depression and living in a high-risk neighborhood influence the occurrence of behavioral problems during childhood and adolescence. Based on a meta-analysis that focused on the association between child depression and parenting, Infurna et al. (2016) report associations between neglect and psychological abuse with the occurrence of depressive symptoms during adolescence and adulthood.

Other studies also verified that maternal depression influences the use of negative rearing practices (Dow-Fleisner, 2018; Silk et al., 2011; Trepat, Granero, & Ezpeleta, 2014; Vafaeenejad, Elyasi, Moosazadeh, & Shahhosseini, 2018; Winsper, Wolke, & Lereya, 2015; Zalewski, Thompson, & Lengua, 2017) and harms marital relationships (Vafaeenejad et al., 2018; Winsper et al., 2015). Additionally, various studies suggest that maternal depression negatively impacts child development (Conners-Burrow et al., 2016), both in terms of internalizing (Ahun et al., 2017; Silk et al., 2011; Zalewski et al., 2017), and externalizing problems (Cilino, Silva-Rodrigues, Pereira-Lima, Pizeta, & Loureiro, 2018; Zalewski et al., 2017). Some of these studies are presented below.

Dow-Fleisner (2018) compared groups of mothers with and without depression, reporting that the depression group presented higher rates of parental stress and lower rates of parental competence, with greater use of psychological and physical aggression. Based on a broad bibliographic review, Vafaeenejad et al. (2018) determined that multiple parental factors and children’s factors influenced parenting styles, while depression and anxiety may contribute to dysfunctional parenting, leading parents to mistreat children, which results in a greater likelihood of using inadequate and inefficacious parenting practices. The authors stress that depression and parental stress may affect marital relationships and interaction between parents and children.

Negative parenting, expressed by maternal behaviors like hostility and resentment, yelling and spanking children, as well as the presence of parental conflict, was addressed in the study conducted by Winsper et al. (2015), who verified that anxiety and pre- and postnatal maternal depression and the use of alcohol were associated with maladaptive parenting. The results show that maladaptive parenting and family adversity mediated associations between prenatal anxiety/depression and borderline personality disorder among children aged 11 and 12 years old.

Zalewski et al. (2017) conducted a longitudinal study to address inconsistent discipline and child rejection by monitoring 214 American families with children aged from 8 to 12 years old for 3 years. The study reports that maternal depressive symptoms were positively correlated with inconsistent discipline and rejection, as well as with children’s internalizing and externalizing problems. The study also highlights that high levels of physical punishment predicted more internalizing and externalizing problems, while a small share of the sample (9%) reported from moderate to high levels of physical punishment.

Ahun et al. (2017) analyzed the effects of parenting and maternal depression symptoms during early childhood in a longitudinal cohort study, reporting that maternal depression symptoms were associated with high levels of internalizing problems, regardless of other maternal factors like quality of parenting, family functioning, and socioeconomic conditions or children’s variables such as sex and temperament. Bi-directional relationships between maternal depression and children’s internalizing problems were addressed in a longitudinal study conducted by Kuckertz, Mitchell, and Wiggins (2018), who verified that psychological aggression was the only parental behavior that mediated this relationship.

In another longitudinal study, Conners-Burrow et al. (2016) addressed a sample of 1844 families whose children were monitored from the age of 14 months up to after the age of 11 years old and found a strong association between early maternal depression and the occurrence of behavioral problems at the age of 11 years old. In the same direction, Trepat et al. (2014) longitudinally addressed a sample of 622 children aged between 3 and 5 years old. The authors concluded that maternal anxiety-depression mediated physical punishment and challenging-oppositional behavior among boys and girls. Silk et al. (2011) addressed a sample of mothers of preschool and school-aged children and also found that maternal depression was associated with negative parenting practices, which predicted internalizing problems among children.

Rovaris and Bolsoni-Silva (2018) addressed a sample of 76 mothers of preschool-aged children, both sexes, and found the following correlations for the sample of preschool-aged children without behavior problems: (a) positive parental interactions were associated with the children’s social skills and with positive marital interactions; (b) negative parental interactions were directly associated with behavioral problems and negative marital interactions; (c) social skills were inversely proportional to behavior problems among girls; and (d) children’s social skills were also directly correlated to behavior problems, while positive parenting practices were directly associated with indicators of behavioral problems, which suggests that parenting practices are used inconsistently.

According to the analysis of reviews considering the relationship between maternal depression, parenting, marital relationship, and child behavior, we verify that (a) maternal depression has been associated with less competent child-rearing practices and an excessive use of negative practices (Dow-Fleisner, 2018; Silk et al., 2011; Pizeta et al., 2013; Trepat et al., 2014; Vafaeenejad et al., 2018; Zalewski et al., 2017), increasing the risk of children developing internalizing and externalizing behavioral problems (Conners-Burrow et al., 2016; Pizeta et al., 2013; Silk et al., 2011; Trepat et al., 2014; Zalewski et al., 2017); (b) mental health conditions such as parental stress and maternal depression may affect marital relationship and interaction with children (Vafaeenejad et al., 2018); (c) the literature diverges in regard to the relationship between maternal depression variables, marital relationships, and children’s behaviors. Ahun et al. (2017) reports that maternal depression predicted internalizing behavioral problems regardless of parenting variables, family functioning, or other sociodemographic variables of both parents and children, while the study by Kuckertz et al. (2018) verified the mutual influence of maternal depression and internalizing symptoms, but only a mediating role for psychological aggression; (d) an inverse relationship was also found between depression and the use of substances in the pre- and postnatal period, due to an association with maladaptive parenting (Winsper et al., 2015); and (e) a relationship was also found between positive practices and children’s social skills and behavior problems, with associations between positive practices and positive marital relationships and between negative practices, negative marital relationships, and behavior problems (Rovaris & Bolsoni-Silva, 2018).

Regardless of the studies’ design, results show that maternal depression impacts child behavior, both internalizing and externalizing problems, but mainly internalizing behaviors. Similarly, associations between negative parenting practices, maternal depression, and behavioral problems seem to be established, as well as between positive parenting practices and child social skills. Parenting practices appear to be associated with marital relationships and together they seem to influence maternal mental health.

As far as this literature review could reach, we found that the presence of maternal depression affects marital relationships and child-rearing practices, while both contribute to children’s behavioral problems. There is, however, a gap in terms of studies controlling for maternal depression as an independent variable and studies addressing marital relationships in bi-parental families in order to investigate the influence of these variables on parenting practices, social skills, and child behavioral problems. Maternal depression is among the mental health variables and is the focus of this study, the objective of which is to understand the mutual influence of maternal depression on parenting practices and marital relationships to better understand the outcome of child behavioral problems.

Knowledge concerning the negative impact of maternal depression on behavior problems during childhood is widely established; however, there is a lack of studies addressing multiple context variables that contribute to such outcomes to identify associations that support the proposal of prevention and intervention practices.

## Objectives

The aim of this study was to verify associations between positive and negative parenting practices, marital relationships, social skills, and behavioral problems among children from bi-parental families with and those without maternal depression; to identify the predictive effect of positive and negative parenting practices, marital relationships, children’s social skills, and maternal depression, for indicators of internalizing, externalizing behavior problems and internalizing and externalizing comorbidities.

## Method

This is a case-control study with a cross-sectional, correlational, and predictive design (Hulley et al., 2008). This type of design is intended to verify whether exposure to a given condition, in the case of this study, indicators of maternal depression, is associated with indicators of behavior problems. In principle, the participants should be drawn from the same population, which is difficult to ensure. One way to verify that is to check whether the groups are homogeneous in terms of sociodemographic variables, which are presented in the characterization of the participants.

### Ethical aspects

This study project was approved by the Institutional Review Board at the university where it was conducted. It is part of a larger project titled *Saúde*, *Habilidades Sociais Conjugais e Educativas Parentais: comparações quanto a escolaridade*, *gênero e problemas de comportamento* [Health, Marital, Social, and Parenting Skills: comparisons between education, gender and behavioral problems] (Process No. 5826/46/01/10).

### Participants

A total of 70 mother-child pairs participated in the study. The mothers had a partner/spouse, not necessarily the child’s biological father, with 35 mothers presenting depression indicators (G1) and 35 not presenting (G2). The children’s variables, such as schooling (whether children were in kindergarten or elementary school) and sex, were controlled for in the composition of the groups, in addition to the number of kindergarten children (*n* = 17) and elementary school children (*n* = 18), as well as the same number of boys (9 in kindergarten and 15 in elementary school, *n* = 24) and girls (8 in kindergarten and 3 in elementary school, *n* = 11). The variable indicators of depression (score ≥ 10 obtained on the PHQ-9 – Patient Health Questionnaire-9) were the criterion used to include women/mothers in the clinical indicators sample (Osório, Mendes, Crippa, & Loureiro, 2009). The means/standard deviations obtained in the clinical group presenting indicators of depression and the non-clinical group on the PHQ-9 concerning indicators of maternal depression were M/SD of the clinical group = 14.00/4.33; M/SD of the non-clinical group = 2.29/2.38.

According to Student’s *t* test and the Chi-square test, no demographic differences concerning the children and mothers were found between the clinical and non-clinical groups: (a) the clinical group was aged 31.76 years old, on average (SD = 5.09), while the non-clinical group was 33.50 years old, on average (SD = 7.25), *p* = 0.261; (b) the children of the mothers in the clinical group were 5.88 years old, on average (SD = 2.52), and children of the mothers in the non-clinical group were 6.29 years old, on average (SD = 2.94), *p* = 0.543; (c) maternal education was divided between up to 8 years of schooling (27 in the clinical group and 26 in the non-clinical group) and more than 8 years of schooling (8 in the clinical group and 9 in the non-clinical group), *X*^2^ = 0.780; (d) income was coded as up to two times the minimum wage (clinical = 15; non-clinical = 12) and more than two times the minimum wage (clinical 20; non-yclinical = 23), *X*^2^ = 0.461; and (e) the sample was also similar in the number of mothers with a paid job and those who were homemakers: 22 mothers in the clinical group presenting indicators of depression worked outside home and 12 did not, while 15 mothers in the non-clinical group had a paid job and 20 did not (*X*^2^ = 0.69).

The assessments of mothers regarding their children’s behaviors using the Child Behavior Checklist – CBCL (Achenbach & Rescorla, 2001) revealed that 21 children did not present indicators of behavior problems; 16 presented only indicators of internalizing problems; 13 presented only indicators of externalizing problems; and 20 presented indicators of internalizing and externalizing problem comorbidities.

### Sampling

This study’s participants were selected from a database of mothers/fathers/caregivers of preschool-aged children (*n* = 121) and school-aged children (*n* = 108), totaling 229 respondents. We identified 49 (25 preschoolers and 23 school-aged children) respondents in this database who presented indicators of depression according to the Patient Health Questionnaire-9 – PHQ-9 (Osório et al., 2009); that is, they presented a score equal to or greater than 10, while 182 respondents presented no depression indicators. The next step was to identify whether the biological mothers were married or in a consensual union, a criterion that excluded 11 participants. (four were single, three divorced, two were not the biological mothers, and two did not report their marital status). Thus, a sample of 38 mothers presenting indicators of depression and in a consensual union remained in the sample. Three participants did not correctly complete the instrument assessing marital relationships (*n* = 2) or the one assessing child behavior (*n* = 1) and were excluded from the sample. The final sample composing the clinical sample consisted of the 35 remaining biological mothers with depression. Afterwards, a non-clinical group (*n* = 35) with the same demographic characteristics of the clinical group, such as the mother being married or in a consensual union, the children’s sexes, and years of schooling, was selected for this case control study from a sample of 182 participants without depression.

### Instruments

Report instruments were use in the data collection and were applied exclusively to mothers.

1. *Roteiro de Entrevista de Habilidades Sociais Educativas Parentais* [Parenting Social Skills Interview Guide] (RE-HSE-P, Bolsoni-Silva, Loureiro, & Marturano, 2016). This instrument is an interview script that assesses (positive and negative parenting practices) and children’s behaviors (behavioral problems and social skills). This semi-structured interview assesses the frequency and diversity of positive parenting practices (also called social parenting skills), contextual variables, negative parenting practices, reports of behavioral problems, and child social skills. Contextual variables assess how diverse the situations are in which the behaviors of parents and children take place, for instance, whether conversations between mother and child that take place at various times of the day refer to subjects that interest the child. This diversity is indicated in the context category. It is possible to obtain a score for positive parenting practices, negative parenting practices, behavioral problems, social skills, and context variables, all variables that were considered in this study. This instrument also measures the frequency of behaviors, such as talking, expressing negative feelings, difficulty keeping promises, marital agreement, complaints regarding the child, and whether the child asks questions about sexuality. Before the specific questions, the following information is collected: level of education, marital status, socioeconomic level, number of children, and whether the mother has a paid job. Factor analysis resulted in two factors: positive and negative aspects of the interaction between parents and children. The alpha for this study’s sample was 0.846. The instrument discriminated between children with and without behavioral problems in a sample from the community, in divorced families, and families with children with language and/or hearing deficits. Roc curve analysis presented an area of 0.769 (*p* = 0.001), discriminating between children with and without indicators of problems according to the CBCL. This instrument provided measures of positive and negative parenting practices, context variables, reports of behavioral problems, and child social skills.
2. *Questionário de Respostas Socialmente Habilidosas para Pais* (*QRSH*-*Pais*) [Questionnaire for Socially Skillful Responses – Parents’ version] assesses the frequency of socially skillful responses according to the reports of fathers and mothers (Bolsoni-Silva, Marturano, & Loureiro, 2011). The QRSH assesses social skills based on a Likert scale. The instrument, with an alpha equal to 0.82, discriminated between children with and without behavioral problems according to the reports of mothers/primary caregivers. A cutoff point of 23 was identified in a psychometric study (submitted paper) to indicate a deficit of social skills. This instrument provided a measure concerning the variable of child social skills.
3. *Questionário de Relacionamento Conjugal – QRC* [Marital Relationship Questionnaire] (Bolsoni- Silva & Marturano, 2010). This instrument has six sets of information that assess, on a Likert scale, the frequency and how positively one describes her spouse; affection that is manifested and received; (positive and negative) communication; identification of behaviors considered positive or negative; and also marital satisfaction. The instrument’s reliability was verified through a test-retest that obtained 0.84 for women and 0.94 for men (Bolsoni- Silva & Marturano, 2010). A psychometric study (submitted paper) analyzed the instrument with a sample of 106 women and 105 men and identified three factors: factor 1 QRC—Total positive (frequency with which spouse receives affection, frequency of appropriate communication, frequency partner does what you like, positively defines spouse, shows affection, receives affection, positive communication, and spouse’s positive behavior); factor 2 QRC—Total negative (frequency with which partner does what you do not like, negatively defines spouse, negative communication, spouse’s negative behavior); factor 3 QRC—marital satisfaction (frequency with which partner manifests affection, assessment of marital relationship), altogether explaining 58.632% of the variance: 30.616% is explained by factor 1, 15.295% by factor 2, and 12.721% by factor 3. The instrument’s total alpha was 0.871. This instrument provided measures of positive marital relationships and negative marital relationships.
4. Child Behavior Checklist – CBCL (Achenbach & Rescorla, 2001) for preschool and school-aged children (4 to 18 years old). This instrument provides indicators of emotional and behavioral problems that can be considered behavioral correlates of psychopathologies. The parents’ reports are provided using a Likert scale that assesses the frequency of 113 statements indicating behavioral problems. The results are organized into internalizing, externalizing, and total problems. Bordin, Mari, and Caeiro (2003) found satisfactory test positivity and morbidity criteria for clinical and non-clinical profiles. This instrument provided a measure of child behavioral problems.
5. Patient Health Questionnaire - 9 (PHQ-9). The questionnaire was proposed and validated by Spitzer, Kroenke, and Williams (1999) and by Kroenke, Spitzer, and Williams (2001) to assess indicators of maternal depression and is a module directly based on diagnostic criteria for Major Depression according to the DSM-5. It enables screening for both current signs and symptoms of major depression, as well as classifying the disorder as mild, moderate, or severe; the higher the score, the more frequently indicators of problems occur. In Brazil, a psychometric study was conducted using the PHQ-9 (Osório et al., 2009) and the structured clinical interview for the DSM-5, which presents excellent validity, was the gold standard. The results found were ROC (AUC) equal to 0.998 (*p* < 0.001) and a cutoff point equal to or greater than 10 was the most appropriate for screening depression, with sensitivity (S) equal to 1.00, specificity (E) equal to 0.98, positive predictive value equal to 0.97, negative predictive value equal to 1.00, and diagnostic efficacy equal to 0.999 (Osório et al., 2009). This instrument provided measures of indicators of maternal depression and allowed the clinical and non-clinical groups to be established.

### Data collection procedures

After approval was obtained from the Child Education Department of a medium-sized city in the interior of São Paulo, Brazil, we contacted Early Childhood, Elementary, and Middle Schools. The principals, teaching coordinators, and teachers received clarification regarding this study’s objectives. The schools were selected considering the city’s extension area so that data would be collected from schools in both the central and peripheral areas. Another criterion used was intended to ensure that there would be the same number of preschool- and school-aged children in each neighborhood, so that both groups of children would be equally represented.

The teachers were asked to nominate two children attending their classes: one they considered to have behavioral problems and one without behavioral problems. The mothers of the nominated children were contacted and invited to participate in the study, and received clarifications about its objective. Both the mothers and teachers who agreed to participate signed free and informed consent forms.

The mothers’ assessments were conducted at a location they found the most convenient (at their homes, schools, or the Applied Psychology Center at the university); the individual meetings lasted approximately one hour and a half.

### Data analysis and treatment procedure

Data were tabulated in the following sequence: (a) the participants were assigned to groups according to presenting indicators of maternal depression or not; (b) the instruments were tabulated according to each instrument’s individual instructions; (c) the variables from the clinical and non-clinical groups for indicators of depression were correlated using Pearson’s test; and (d) numerical variables were selected for the simple regression linear analysis was then performed, in which having indicators of behavioral problems, only internalizing or only externalizing problems, or internalizing/externalizing comorbidities, were the dependents variables. Correlations were classified according to Primi, Muniz, and Nunes (2009) as weak (0–0.25), moderate (0.26–0.50), strong (0.51–0.70), or very strong (≥ 0.71).

## Results

Table 1 presents the correlations for the groups with and without indicators of depression and Table 2 presents the correlations for the total sample (G1 + G2). Tables 3, 4, and 5, respectively, present the results of the simple linear regression analysis considering the different independent variables assessed for indicators of internalizing, externalizing problems, and internalizing/externalizing problems comorbidities.

**Table 1:** Correlations between maternal depression, parenting, marital interaction, and child behavior for G1 (*n* = 35) and G2 (*n* = 35)

Mother	Infant G1			Infant G2		
	Child social skills—QRSH	Child social skills—RE-HSE-P	Reports of behavioral problems—RE-HSE-P	Child social skills—QRSH	Child social skills—RE-HSE-P	Reports of behavioral problems—RE-HSE-P
	r (p value)	r (p value)	r (p value)	r (p value)	r (p value)	r (p value)
Maternal depression clinical score-PHQ-9	0.01 (0.970)	− 0.19 (0.263)	0.12 (0.511)	0.54 (0.001)**	− 0.09 (0.583)	0.34 (0.047)
Positive parenting–RE-HSE-P	0.01 (0.945)	0.60 (0.000)**	− 0.06 (0.709)	0.19 (0.283)	0.28 (0.107)	− 0.04 (0.800)
Diversity of context of interactions– RE-HSE-P	0.07 (0.687)	0.32 (0.056)	0.08 (0.647)	0.19 (0.267)	0.01 (0.556)	− 0.15 (0.376)
Negative parenting– RE-HSE-P	− 0.04 (0.824)	0.03 (0.853)	0.05 (0.002)**	0.09 (0.621)	0.37 (0.028)*	0.51 (0.002)**
Factor 1 QRC—Total positive	0.11 (0.530)	0.09 (0.602)	− 0.08 (0.626)	0.26 (0.128)	0.27 (0.121)	− 0.16 (0.366)
Factor 2 QRC—Total negative	− 0.10 (0.558)	0.09 (0.605)	− 0.12 (0.479)	− 0.32 (0.058)	− 0.13 (0.464)	0.33 (0.50)*
Factor 3 QRC—marital satisfaction	− 0.21 (0.227)	− 0.32 (0.059)	0.16 (0.351)	− 0.27 (0.114)	0.06 (0.735)	0.15 (0.397)

*The correlation is significant at the 0.05 level (2 extremities)

**The correlation is significant at the 0.01 level (2 extremities)

*QRSH*, *PHQ-9*, *RE-HSE-P*, *QRC* = acronyms of instruments

**Table 2:** Correlations between maternal depression, parenting, marital interaction and child behavior for to the total sample (*n* = 70; G1 + G2)

Mother	Infant G1 + G2 (*n* = 70)		
	Child social skills—QRSH	Child social skills—RE-HSE-P	Reports of behavioral problems—RE-HSE-P
	*r* (*p* value)	*r* (*p* value)	*r* (*p* value)
Maternal depression clinical score-PHQ-9	− 0.18 (0.128)	− 0.04 (0.728)	0.09 (0.444)
Positive parenting–RE-HSE-P	0.09 (0.444)	0.43 (0.000)**	− 0.05 (0.671)
Diversity of context of interactions–RE-HSE-P	0.12 (0.342)	0.21 (0.076)	− 0.04 (0.726)
Negative parenting– RE-HSE-P	− 0.03(0,785)	0.21(0.79)	0.49 (0.000)**
Factor 1 QRC—Total positive	0.19 (0.109)	0.12 (0.309)	− 0.10 (0.393)
Factor 2 QRC—Total negative	− 0.21 (0.081)	0.03 (0.817)	0.08 (0.534)
Factor 3 QRC—marital satisfaction	− 0.17 (0.164)	− 0.19 (0.107)	0.14 (0.242)

*The correlation is significant at the 0.05 level (2 extremities)

**The correlation is significant at the 0.01 level (2 extremities)

*QRSH*, *PHQ-9*, *RE-HSE-P*, *QRC* = acronyms of instruments

**Table 3:** Simple linear regression analysis with only internalizing behavior problems as the dependent variable (*n* = 16, and non-clinical—*n* = 21) and the following independent variables: maternal depression, positive parenting practices, negative parenting practices, quality of marital relationship, children’s social skills, and mothers’ reports of problems

	*B*	*β*	*T*	p*	CI (95%)	Total adjusted *R*^2^
Internalizing problems						
Maternal depression–Clinical score-PHQ-9	0.014	0.156	0.935	0.356	− 0.016–0.045	− 0.003
Positive parenting–RE-HSE-P	0.016	0.163	0.979	0.334	− 0.018–0.050	− 0.001
Negative parenting–RE-HSE-P	0.014	0.105	0.624	0.537	− 0.031–0.058	− 0.017
Factor 1 QRC—Total positive	− 0.003	0.007	− 0.418	0.679	− 0.018–0.012	− 0.023
Factor 2 QRC—Total negative	0.021	0.295	1.829	0.076	− 0.002–0.044	0.061
Factor 3 QRC—marital satisfaction	− 0.028	− 0.029	− 0.172	0.865	− 0.362–0.306	-0.028
Child social skills—QRSH	− 0.024	− 0.287	− 1.772	0.085	− 0.051–0.003	0.056
Report of behavioral problems—RE-HSE-P	0.005	0.039	0.233	0.817	− 0.041–0.051	− 0.027

**Table 4:** Simple linear regression analysis with only externalizing behavior problems as the dependent variable (*n* = 13, and non-clinical—*n* = 21) and the following independent variables: maternal depression, positive parenting practices, negative parenting practices, quality of marital relationship, children’s social skills, and mothers’ reports of problems

	*B*	*β*	*T*	p*	CI (95%)	Total adjusted *R*^2^
Maternal depression–Clinical score-PHQ-9	0.084	0.558	3.803	**0.001**	0.039–0.129	0.290
Positive parenting–RE-HSE-P	− 0.023	− 0.107	− 0.610	0.546	− 0.099–0.054	− 0.019
Negative parenting–RE-HSE-P	0.043	0.141	0.806	0.426	− 0.065–0.151	− 0.011
Factor 1 QRC—Total positive	− 0.032	− 0.418	− 2.604	**0.014**	− 0.057 to − 0.007	0.149
Factor 2 QRC—Total negative	0.053	0.376	2.293	**0.029**	0.006–0.099	0.114
Factor 3 QRC—marital satisfaction	− 0.399	− 0.425	− 2.656	**0.012**	− 0.706 to − 0.093	0.155
Child social skills—QRSH	− 0.055	− 0.164	− 0.942	0.353	− 0.174–0.064	− 0.003
Report of behavioral problems—RE-HSE-P	0.034	0.140	0.798	0.431	− 0.052–0.119	− 0.011

**Table 5:** Simple linear regression analysis with internalizing and externalizing comorbidities as the dependent variable (*n* = 13, and non-clinical—*n* = 21) and the following independent variables: maternal depression, positive parenting practices, negative parenting practices, quality of marital relationship, children’s social skills, and mothers’ reports of problems

	*B*	*β*	*T*	p*	CI (95%)	Total adjusted *R*^2^
Maternal depression–Clinical score-PHQ-9	0.104	0.521	3.808	**0.000**	0.049–0.159	0.252
Positive parenting–RE-HSE-P	− 0.099	− 0.350	− 2.336	**0.025**	− 0.185 to − 0.013	0.100
Negative parenting–RE-HSE-P	0.173	0.509	3.695	**0.001**	0.078–0.268	0.240
Factor 1 QRC—Total positive	− 0.029	− 0.462	− 3.252	**0.002**	− 0.047 to − 0.011	0.193
Factor 2 QRC—Total negative	0.063	0.353	2.355	**0.024**	0.009–0.117	0.102
Factor 3 QRC—marital satisfaction	− 0.006	− 0.003	− 0.019	0.985	− 0.620–0.609	− 0.026
Child social skills—QRSH	− 0.106	− 0.428	− 2.958	**0.005**	− 0.178 to − 0.034	0.162
Report of behavioral problems—RE-HSE-P	0.142	0.398	2.713	**0.010**	0.036–0.248	0.137

As shown in Table 1, positive parenting practices were strongly correlated with child social skills. Negative parenting practices were positively and strongly correlated with reports of behavioral problems.

Relationships were also found in the clinical group of mothers. The score for indicators of maternal depression was moderately associated with a negative marital relationship (*r* = 0.35, *p* = 0.038*), while positive parenting practices were moderately correlated with context variables (*r* = 0.43, *p* = 0.01*).

Indicators of depression in the non-clinical group of mothers (subclinical scores) were positively associated with reports indicators of behavioral problems and inversely correlated with child social skills. Reports indicators of behavioral problems were directly associated with maternal depression and negative marital relationships, and negative parenting practices were associated with child social skills and negative parenting practices. Reports indicators of behavioral problems, indicating that parenting practices are somewhat inconsistent.

Other relationships were found in the non-clinical group of mothers: indicators of depression were inversely correlated with positive marital relationships (*r* = − 0.40, *p* = 0.016*); reports indicators of behavioral problems were inversely related to scores concerning child social skills (*r* = − 0.48, *p* = 0.004**); positive parenting practices were directly associated with contextual variables (*r* = − 0.46, *p* = 0.005**), which refer to the diversity of positive interactions between mothers and children. An inverse relationship was found between positive marital relationship, negative marital relationship (*r* = − .64, *p* = 0.000**), and marital satisfaction (*r* = − 0.38, *p* = 0.032*). Most correlations were moderate.

Table 2 presents relationships among parenting behaviors, marital relationship, and children’s behaviors. As expected, negative parenting practices were directly associated with reports of behavioral problems, while positive parenting practices were associated with child social skills.

Relationships between different repertoires for mothers and children were also found. Positive parenting practices were associated with diversity of interactions (*r* = − 0.45, *p* = 0.000**) and positive marital relationships (*r* = 0.29, *p* = 0.014*). The reports indicators of problems were inversely related to child social skills (*r* = − 0.27, *p* = 0.023*). Indicators of maternal depression were negatively associated with positive practices (*r* = − 0.30, *p* = 0.011*) and positive marital relationships (*r* = − 0.50, *p* = 0.000**) and directly related to negative marital relationships (*r* = − 0.59, *p* = 0.000**). Positive marital relationships were inversely related to negative marital relationships (*r* = 0.56, *p* = 0.000**). Therefore, a direct relationship was found between parenting practices and child behaviors, between marital relationships and maternal depression, as well as between depression indicators and positive parenting practices.

Table 3 shows that none of the independent variables in the simple linear regression predicted indicators of internalizing behavior problems.

Both indicators of maternal depression and marital relationship appeared as significant independent variables for indicators of externalizing problems in the simple linear regression for the three factors (total positive, total negative, and marital satisfaction).

According to Table 5, indicators of internalizing and externalizing comorbidities were influenced by multiple independent variables: maternal depression, positive and negative parental practices, positive and negative marital relationships, children’s social skills, and behavioral problems reported by mothers.

## Discussion

This study’s objective was to identify associations between multiple variables, such as maternal depression, parenting, marital relationship, social skills, and reports of indicators of emotional and behavioral problems that can be considered behavioral correlates of psychopathologies, measured by the CBCL.

A case control design was adopted and some sociodemographic variables were controlled in both groups. Only bi-parental families were included because that is a variable that influences child behavior and parental practices (Cerveira, 2015; Crestani et al., 2013). It is known that merely having a bi-parental family does not ensure good child development, considering the complexity of conditions involved in a family environment, such as (a) problematic parental practices (Dow-Fleisner, 2018; Hameister et al., 2015; Silk et al., 2011; Trepat et al., 2014; Vafaeenejad et al., 2018; Winsper et al., 2015; Zalewski et al., 2017), (b) difficult marital relationships (Vafaeenejad et al., 2018; Winsper et al., 2015), and (c) mental health problems faced by parents (Ahun et al., 2017; Cilino et al., 2018; Silk et al., 2011; Zalewski et al., 2017) and mental health problems experienced by children (Winsper et al., 2015).

A case-control design was adopted in this study to match some sociodemographic and clinical variables known to influence child behavior, such as sex and level of education (Alvarenga et al., 2012; Pizeta et al., 2013); variables were matched in the groups with and without depression. Maternal sociodemographic variables, such as income, education, age, and whether they had a paid job, were compared between the non-clinical and clinical groups and the samples were found to be homogeneous. In this study, indicators of maternal depression were identified using PHQ-9 and was the criterion used to organize the groups; thus, it was a controlled independent variable, considering that the levels of mothers’ depressive symptoms differently impact child behavior (O’Connor, Langer, & Tompson, 2017). The analysis in which variables are controlled enabled establishing relationships between the variables of interest.

Correlations between the scores concerning the indicators of depression and difficulties in marital relationships were found in the group with depression, a finding that agrees with the results reported by other studies (Hsiao, 2017; Najman et al., 2014; Neves & Duarte, 2015). Positive parenting practices were strongly associated with children’s social skills and contextual variables (Rovaris & Bolsoni-Silva, 2018). Note that positive practices were also identified in the clinical group despite difficulties faced with depression, suggesting the presence of resources to be used in interventions. Positive parenting practices are a protective factor and promote child social skills (Rovaris & Bolsoni-Silva, 2018), a piece of information corroborated here; these variables appear correlated in this study. As expected, negative parenting practices were strongly associated with reports of behavioral problems, an association that was identified in both groups, regardless of the presence of maternal depression, which agrees with the findings reported by other studies (Dow-Fleisner, 2018; Silk et al., 2011; Pizeta et al., 2013; Rovaris & Bolsoni-Silva, 2018; Trepat et al., 2014; Vafaeenejad et al., 2018; Zalewski et al., 2017). Note, however, that the scores concerning the indicators of depression obtained by the sample of mothers with indicators of depression were not associated with the indicators of children’s behaviors, which seems to disagree with the literature (Conners-Burrow et al., 2016; Pizeta et al., 2013; Silk et al., 2011; Trepat et al., 2014; Zalewski et al., 2017). In the total sample, maternal depression was inversely proportional to positive practices (Dow-Fleisner, 2018; Silk et al., 2011; Pizeta et al., 2013; Trepat et al., 2014; Vafaeenejad et al., 2018; Zalewski et al., 2017) and directly related to children’s social skills (Rovaris & Bolsoni-Silva, 2018), which in turn were inversely proportional to indicators of behavioral problems (Rovaris & Bolsoni-Silva, 2018). Therefore, indicators of maternal depression indirectly influence parenting and child behavior.

Some correlations identified in the non-clinical group also occurred in the sample of mothers without depression. In this group, scores of indicators of depression were inversely associated with positive marital relationships and directly associated with negative marital relationships, supporting the statement from Féres-Carneiro and Neto (2010) that the quality of a marital relationship is essential to the health of a couple and family. Depression scores were directly associated with reports indicators of behavioral problems (Pizeta et al., 2013; Conners-Burrow et al., 2016; Infurna et al., 2016;Shaw et al., 2016) and inversely associated with child social skills (Pizeta et al., 2013). These results seem to indicate that the mood of mothers influences interactions with children even in the sample of mothers without depression.

In this non-clinical sample, reports indicators of behavioral problems were inversely related to the children’s social skills (Rovaris & Bolsoni-Silva, 2018) and directly associated with maternal depression (Conners-Burrow et al., 2016; Infurna et al., 2016; Shaw et al., 2016). Indicators of behavioral problems were associated with negative marital relationships (Fantinato & Cia, 2015; Hameister et al., 2015; Ramirez Castillo, 2007; Rhoades, 2008) and the use of negative parenting practices (Silk et al., 2011). As expected, positive parenting practices were associated with contextual variables (Rovaris & Bolsoni-Silva, 2018). Additionally, marital relationships and parenting were directly associated in the total sample (Kanoy et al., 2003; Krishnakumar & Buehler, 2000; Rovaris & Bolsoni-Silva, 2018), while positive parenting was inversely proportional to the occurrence of maternal depression (Pizeta et al., 2013). Relationships between the mothers’ different repertories are highlighted, in which positive practices were associated with positive marital relationships and negative practices with negative marital relationships, in addition to an inverse direction between positive and negative marital relationships and marital satisfaction, which seems to agree with the literature (Hameister et al., 2015; Rovaris & Bolsoni-Silva, 2018). Depression, in turn, was inversely associated with positive marital relationships and directly associated with negative marital relationships, suggesting once again that maternal mental health influences marital relationships (Hsiao, 2017; Najman et al., 2014; Neves & Duarte, 2015).

Some relationships were expected but not found, such as (a) scores concerning indicators of depression were not associated with negative parenting practices, which is contrary to what is reported in the literature (Pizeta et al., 2013; Trepat et al., 2014) and (b) positive parenting was not inversely related to behavioral problems (Rovaris & Bolsoni-Silva, 2018). These divergences may be a result of the method used, such as the case control design, in which the group with indicators of depression had a counterpart that did not exhibit depression; the groups were composed of bi-parental families with homogeneous conditions, such as the instruments used to collect data and measure children’s behaviors, marital relationships, and parenting practices.

The sample was divided by type of behavioral problem in the simple linear regression analysis and indicated the influence of indicators of maternal depression for externalizing problems and for internalizing and externalizing comorbidities, which also agrees with the literature (Ahun et al., 2017; Cilino et al., 2018; Conners-Burrow et al., 2016, Silk et al., 2011; Zalewski et al., 2017), however contrasts in regard to internalizing problems, as no relationship was found between indicators of maternal depression and indicators of internalizing problems (Ahun et al., 2017; Cilino et al., 2018; Silk et al., 2011; Zalewski et al., 2017). Such a divergence may be due to the characteristics of the studies’ samples in regard to size and the way participants were identified and grouped. On the other hand, indicators of maternal depression and positive and negative marital relationships remained in the model for indicators of externalizing problems and indicators of behavior problems in internalizing and externalizing comorbidities, as well as for parental practices (positive and negative) and children’s behaviors (problem behavior and social skills). These results confirm that child behavior problems are multi-determined (Alvarenga et al., 2012), and they are one of the main complaints motivating parents to seek psychological assistance, which if left untreated, may become a chronic condition in the future (Goodman et al., 2011). Thus, paying attention to associated risks is important (Alvarenga et al., 2012; Dow-Fleisner, 2018; Pizeta et al., 2013; Shaw et al., 2016; Vafaeenejad et al., 2018; Winsper et al., 2015).

This study’s findings corroborate the findings reported by other studies to some degree, considering that child repertoire (child social skills and reports indicators of problems) and context variables, which refer to positive and negative social interactions, also predicted behavioral problems. The fact that the groups were matched in regard to the presence or absence of indicators of depression and children of different ages were also included in the same sample may have influenced the results when compared to other studies.

## Conclusions

This study identified strong and moderate associations between parenting and child behaviors. Indicators of maternal depression were correlated with indicators of child behavior and also with marital relationships and parenting.

In general, the findings show that, regardless of the sample (with depression, without depression, or total sample), positive parenting was associated with child social skills and negative parenting was associated with indicators of behavioral problems, indicating the strength of parenting practices in influencing the occurrence of children’s skillful or indicators of problem behaviors. Both the sample without indicators of depression and the total sample revealed an inverse relationship between child social skills and reports indicators of problems, indicating that the acquisition of a skillful repertoire supports decreased behavioral problems. It appears that children learn such skills when mothers use positive parenting practices and diversify their use (context variables). Positive parenting practices in the total sample, which is balanced in terms of mothers with and without maternal depression, were associated with positive marital interactions, suggesting that an improvement in positive parenting practices may also favor positive marital relationships.

Indicators of depression in the total sample and in a sub-sample were associated with marital relationships; higher scores were found for negative marital interaction and lower scores for positive marital relationships. Negative marital interactions were also associated with reports indicators of problems in the group without depression. These findings suggest that interventions seeking to improve marital interactions may support decreased levels of depression, as well as fewer negative marital interactions, which directly or indirectly influence the behavior of children. The higher the number of associated indicators of behavior problems, the higher the number of related independent variables, including positive and negative parenting practices, positive and negative marital relationships, and children’s social skills and complaints of problems. Therefore, the results suggest that there is a bidirectional influence between indicators of child behaviors and indicators of maternal behaviors. This study’s strength derives from the fact that the variables were rigorously controlled and resulted in homogeneous groups, only differing in terms of maternal depression indicators, which allowed marital relationships, parenting practices, and their influence on children’s behavior to be assessed. Nonetheless, the sample is small, which does not allow for generalizations. Further studies with more robust samples, controlling for children’s education and focusing on school-aged children, a time when indicators of behavioral problems become more apparent, may better explain the findings that diverge from the literature. It highlights also that the instruments used for the composition of PHQ-9 and CBCL groups provide problem indicators, not allowing the formulation of depression clinical diagnoses and neurodevelopment disorders, which may be the object of further studies.

This study provides results that suggest potential mutual relationships between maternal depression, parenting, marital relationships, and child behavior; however, longitudinal studies could better clarify these relationships. Another limitation involves the fact that mothers were the only informants and the sample was small; in the future, assessments from teachers, fathers, and the children themselves are desirable, to ensure a multi-informant assessment.

Even though the study combined interviews and scales/inventories to measure indicators of behavior problems and social skills, future studies are needed to combine the use of direct observation measures and other easy-to-apply brief instruments to measure marital conflicts and problem-solving capacity, for instance. Future studies are suggested to adopt instruments with varied characteristics and address multiple informants coexisting with children in different contexts such as family and school (multi-informant and multi-modal measures). The objective is to minimize the impact of a potential influence of the mothers’ depressive symptoms on the report of children’s behaviors. Regarding the maternal depression construct, a renowned and widely used screening instrument with validity recognized in many countries, in line with this study’s objectives, was adopted to measure signs and symptoms of depression in a sample from the community. In future studies, however, an assessment instrument for diagnosing depression would enable to address, in addition to current symptoms, temporal aspects related to the chronicity and severity of symptoms, aspects not considered in this study. It is important to conduct early investigations with parents and children in clinical practice with children in order to prevent and/or for early intervention of behavioral problems.

A longitudinal study with these children in order to monitor the impact on learning would also be interesting to be carried out in future studies. The conclusion is that this study’s results confirm the relevance of intervention programs that include orientation directed to parents, couples’ therapy, and social skills training provided to children as strategies to can minimize behavioral problems and expand the repertoire of children’s social skills and improve the mental health of mothers.

## Data Availability

This study’s data are available upon request made to the primary author.
